# Differential adaptation to multi-stressed conditions of wine fermentation revealed by variations in yeast regulatory networks

**DOI:** 10.1186/1471-2164-14-681

**Published:** 2013-10-04

**Authors:** Christian Brion, Chloé Ambroset, Isabelle Sanchez, Jean-Luc Legras, Bruno Blondin

**Affiliations:** 1 INRA, UMR1083 Science pour l’Œnologie, 2 Place Viala, Montpellier F-34060, France; 2 Montpellier SupAgro, UMR1083 Science pour l’Œnologie, 2 Place Viala, Montpellier F-34060, France; 3 Université Montpellier 1, UMR1083 Science pour l’Œnologie, 2 Place Viala, Montpellier F-34060, France

**Keywords:** Wine yeast, Fermentation, QTL, Transcriptome, Partial disomy, Detoxification

## Abstract

**Background:**

Variation of gene expression can lead to phenotypic variation and have therefore been assumed to contribute the diversity of wine yeast (*Saccharomyces cerevisiae*) properties. However, the molecular bases of this variation of gene expression are unknown. We addressed these questions by carrying out an integrated genetical-genomic study in fermentation conditions. We report here quantitative trait loci (QTL) mapping based on expression profiling in a segregating population generated by a cross between a derivative of the popular wine strain EC1118 and the laboratory strain S288c.

**Results:**

Most of the fermentation traits studied appeared to be under multi-allelic control. We mapped five phenotypic QTLs and 1465 expression QTLs. Several expression QTLs overlapped in hotspots. Among the linkages unraveled here, several were associated with metabolic processes essential for wine fermentation such as glucose sensing or nitrogen and vitamin metabolism. Variations affecting the regulation of drug detoxification and export (*TPO1*, *PDR12* or *QDR2*) were linked to variation in four genes encoding transcription factors (*PDR8*, *WAR1*, *YRR1* and *HAP1*). We demonstrated that the allelic variation of *WAR1* and *TPO1* affected sorbic and octanoic acid resistance, respectively. Moreover, analysis of the transcription factors phylogeny suggests they evolved with a specific adaptation of the strains to wine fermentation conditions. Unexpectedly, we found that the variation of fermentation rates was associated with a partial disomy of chromosome 16. This disomy resulted from the well known 8–16 translocation.

**Conclusions:**

This large data set made it possible to decipher the effects of genetic variation on gene expression during fermentation and certain wine fermentation properties. Our findings shed a new light on the adaptation mechanisms required by yeast to cope with the multiple stresses generated by wine fermentation. In this context, the detoxification and export systems appear to be of particular importance, probably due to nitrogen starvation. Furthermore, we show that the well characterized 8–16 translocation located in *SSU1*, which is associated with sulfite resistance, can lead to a partial chromosomic amplification in the progeny of strains that carry it, greatly improving fermentation kinetics. This amplification has been detected among other wine yeasts.

## Background

Since the development of wine-making, wine yeasts have undergone a specific pattern of evolution and have become highly effective in the fermentation of grape juice [1]. They are able to withstand various stresses, such as low pH or high levels of ethanol. These yeasts are also resistant to inhibitors, such as sulfites [2] and medium-chain fatty acids [3], and are able to continue fermentation for long periods of nutrients starvation. In addition, commercial wine yeasts make a specific contribution to the aroma bouquet of the wine. Knowledge of the genomic bases of these specific features is a prerequisite for understanding the mechanisms underlying adaptation [1,4,5]. Such knowledge would also provide a basis for the improvement of industrial wine yeast strains [6]. Various studies have enhanced our understanding of genomic structural variation in wine yeast strains. Genome sequencing has led to the identification of genomic rearrangements and mutations specific to wine yeasts (reviewed in [7-9]). One of the best examples is the translocation between chromosomes 8 and 16 described by Pérez-Ortín *et al.*[10] leading to overexpression of the sulfite exporter gene *SSU1* and to the resistance of higher levels of sulfite. Dunn *et al.*[11,12] have shown, using comparative genomic hybridization (aCGH), that copy-number variations for several groups of genes (involved in drug responses and ion sensors) are also characteristic of wine strains. Based on an analysis of the wine yeast EC1118 sequence, Novo *et al.*[13] showed that three loci widely conserved in wine strains had been gained by horizontal transfer events from non *Saccharomyces* yeast [14]. Comparison of sequenced genomes has suggested that nucleotide polymorphisms are the major source of phenotypic diversity [15,16]. However the relationships between such genetic variations and phenotypic diversity remain unclear, particularly in the context of alcoholic fermentation.

Quantitative trait locus (QTL)-based approaches are widely used in yeast, to link allelic variations to phenotypic diversity [17-20]. This approach has been applied to wine strains to identify the origin of fermentation traits. Marullo et al. [21] used this approach to show that mutations of the asparaginase gene *ASP1* underlie differences in acetic acid production between two wine strains. QTL-based approaches have proved efficient for identifying the traits associated with a single mutation, but most fermentation traits are under complex polygenic control which is much more difficult to resolve. Using new approaches based on the analysis of very large populations of segregants; Extreme QTL mapping (X-QTL); Ehrenreich *et al.*[22,23] demonstrated the feasibility of identifying the genetic determinants of complex phenotypes, such as drug resistance.

Trait variation in may also results from changes in gene expression of the underlying regulatory networks. Brem *et al.*[24] have demonstrated that regulatory variations could be linked to genomic alterations by QTL approaches. Searches for expression QTLs (eQTLs) are now widely used in yeast to decipher regulatory networks variations [25-29], reviewed in [30,31].

Such approaches can be used to elucidate the origin of regulatory network variations and their impact on fermentation traits, such as the fermentation duration, the nitrogen consumption and the metabolite production. We previously generated a segregating population by crossing the lab strain S288c and a spore derivative of the wine yeast EC1118, for eQTL linkage analysis [4]. One of the main results of this previous study was the detection of a large impact of the *ABZ1* (encoding para-amino benzoate (PABA) synthase) allele on nitrogen assimilation, resulting in large variations in the fermentation rate [4]. Furthermore, a recent QTL study using this lineage implicated *ABZ1* allele variation in aromatic compounds production [32] probably due to the involvement of this gene in amino-acid metabolisms.

We report here of the results of a new search for fermentation traits QTL and eQTL with an enlarged segregant population in the late stages of alcoholic fermentation, more relevant conditions for addressing the stress response. The use of this approach provided us with a broad view of expression variation in alcoholic fermentation and allowed us to identify the genetic origin of variation for several regulatory networks involved in key processes, such as detoxification and sulfate assimilation. An unexpected result of this study was the finding that fermentation rate was controlled by a partial disomy of chromosome 16, revealing a new role in wine yeasts for a well known translocation.

## Results

### Phenotypics and transcriptomic analysis of the 59A×S288c lineage

**Figure 1:**
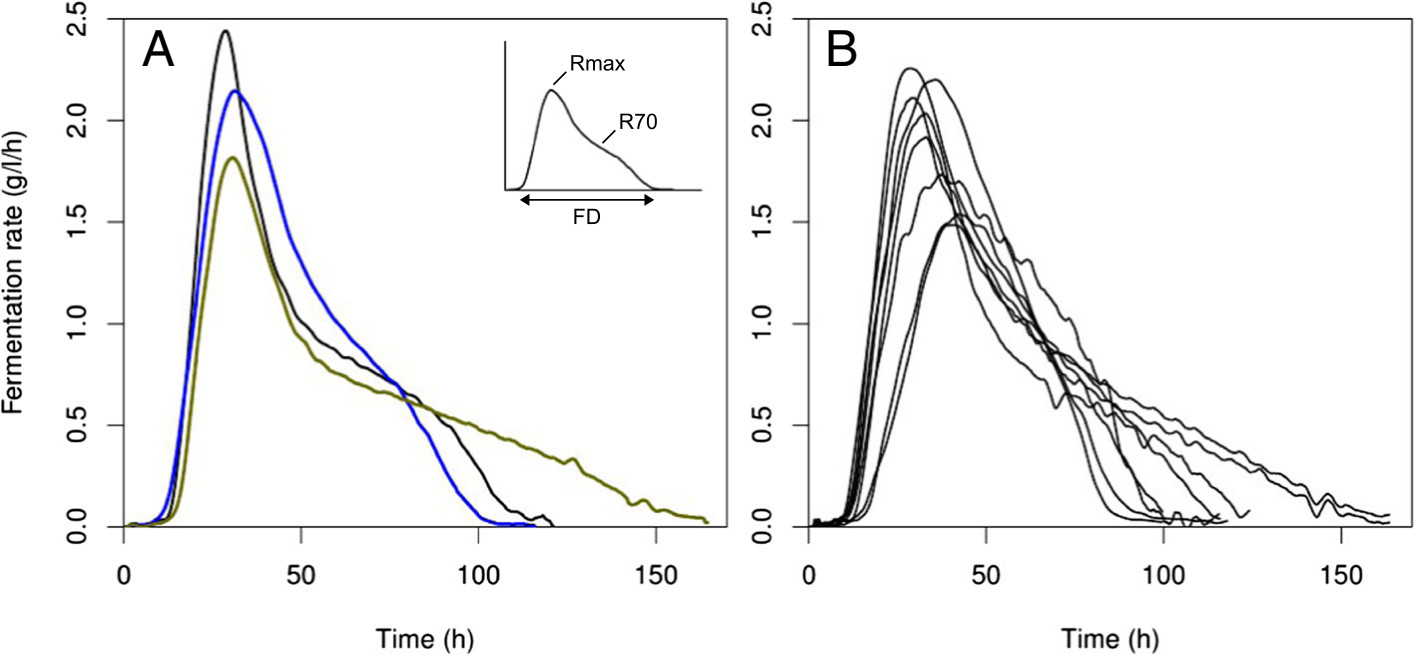
**Fermentation profiles for parental strains and for segregants. (A)** Fermentation profile for the two parental strains and for 59A×S288c: 59A in blue, S288c in green, 59A×S288c in black. **(B)** Fermentation profile for 8 segregants. Sugar was completely consumed for all segregants.

We phenotyped 44 segregants obtained from a cross between the laboratory strain S288c and the wine yeast derivative 59A (as described in Methods). Fermentations were performed in a synthetic medium simulating a grape must (SM425) and containing para-amino benzoate (PABA) to counteract the effect of the *ABZ1* allele [4]. Our analysis were performed in more stringent conditions than that of Ambroset *et al.*[4]: the amounts of ergosterol and oleic acid used were half those generally used (the final concentrations were 7.5 mg/l for ergosterol and 2.5 μl/l for oleic acid) and fermentations were performed at 24°C. We estimated the kinetic properties regarding the lag time and the fermentation rate at 2 stages of the fermentation: Rmax (maximal fermentation rate) and R70 (fermentation rate at 70% of fermentation). The fermentation kinetics of parental strains and of several segregants are shown in Figure 1. Population size and metabolite quantities were determined at the end of the fermentation.

We first checked that phenotypes affected by *ABZ1* allele, such as Rmax, were corrected by the addition of PABA and independent of the allelic form (data not shown). Most of the phenotypes had a high heritability (80% to 97%), indicating that genetic variations had a major impact on overall variations. The dry weight was the only phenotype displaying a low heritability (>50%) and was disregarded. R70 values followed a bimodal distribution, suggesting that the phenotype is mainly controlled by one locus. The other phenotypes, such as Rmax and the amounts of metabolites followed continuous distributions, indicating a probable polygenic control (Additional file 1).

Transcriptome profiles were obtained at 70% of fermentation progress (66 g/l CO_2_ released), corresponding to late stationary phase, 20 to 40 hours after the end of the growth phase (depending on the segregants). At this point in the fermentation, the yeast is subject to nutrient starvation and ethanol stress (8% v/v). We compared RNA abundance between segregants by Agilent mono-color labeling and hybridization on oligonuclotides microarrays as described in Methods. We identified 1610 genes differentially expressed between 59A and S288c with a log_2_ of fold-change (LogFC) of more than 0.7 in either direction (adjPv<0.01).

We assessed the correlation between the fermentation rate (R70) and gene expression and found large sets of genes positively correlated (347 genes involved in cell trafficking and nitrogen reutilization; Funspec [33]) and negatively correlated (351 genes involved in oxidative stress and respiration; Funspec).

### Clustering analysis revealed a partial disomy of chromosome 16 affecting genes expression

A clustering analysis of the whole gene expression dataset revealed significant associations (Pearson correlation coefficient ≥ 0,84) for 887 genes (Additional file 2). Most of the gene clusters obtained were enriched in functional classes, such as mating, mitochondrial translation, ribosomal proteins, ergosterol synthesis and sulfur assimilation consistent with the known coordination of expression of these genes. Some clusters also corresponded to genes correlated with phenotypes (R70, Rmax). Other clusters included physically associated genes (ASP cluster, regions A B and C [13], telomeric genes).

**Figure 2:**
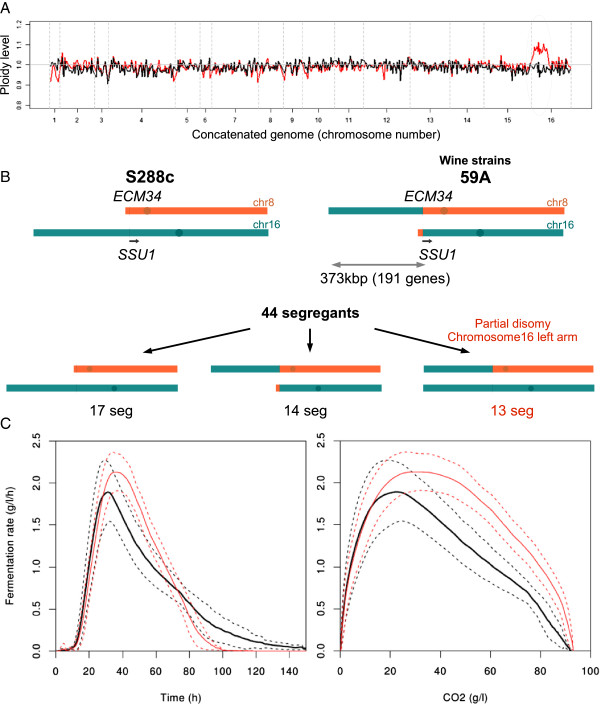
**The 8–16 translocation and impact on fermentation. (A)** CGH profile for the segregant 7a (in red) which has the partial disomy, in comparison to S288C CGH profile (in black). **(B)** Parental and daughter genotype: Crossing 59A which has the translocation with S288c gives four types of segregants: without translocation (S288c like), with the translocation (59A like), with two chr16 left arm (partial disomy) or without chr16 left arm but this combination is not viable, due to the loss of essentials genes (e.g. *NSL1*, *NAB3*, *RPL5*). **(C)** Average fermentation profile for the normal segregants (black) and disomic segregants (red). The doted lines represent standard error. Kinetics are represented in function of time (left) or of CO_2_ release (right).

We identified a cluster of 37 genes displaying a higher expression in 13 segregants. These genes were physically linked and all were located in the first 373 kbp at the start of chromosome 16 (Additional file 2, cluster “chr16 left-arm genes”). A careful mining of the CGH data (from the Affymetrix chip signals) revealed that these 13 segregants carried a 373 kbp duplicated region on the left arm of chromosome 16 (Figure 2A). No other chromosomal aberration was observed. This partial disomy resulted from the translocation of an arm of chromosome 16 onto the chromosome 8 originating from the wine yeast strain, in addition to the presence of a standard chromosome 16 (Figure 2B). This 8–16 translocation is known to be responsible for higher level of sulfite resistance and was found in many wine yeasts [10]. We examined the impact of this partial disomy on transcriptome profiles by comparing the expression data for the 13 disomic segregants with those for the other 31 segregants. As expected, the genes of the duplicated region were more strongly expressed in segregants carrying the disomic region (155 of the 191 genes in the area concerned, adjPv<0.05), consistent with the doubling of gene copy number. We also observed differences in expression for 1305 other genes (adjPv<0.01) not located in this area. The upregulated genes include a high proportion of genes involved in methionine biosynthesis (17 genes, Pv=10^-9^ -FunSpec [33]) whereas the down-regulated genes included a high proportion of genes involve in the oxidative stress response (20 genes, Pv=10^-6^ -FunSpec) and aerobic respiration (37 genes, Pv=10^-14^ -FunSpec). The pattern of functional enrichment of the deregulated genes suggests that disomic strains are less affected by stresses of alcoholic fermentation conditions. Consistent with this hypothesis, we observed that the partial disomy had a major impact on fermentation kinetics (Figure 2C). Partial disomy was associated with significantly higher fermentation rates, a shorter duration of fermentation (*t*-test Pv<10^-6^) and an absence of sluggish fermentation profiles. Levels of acetic acid production were also lower for the disomic segregants (data not shown, *t*-test Pv<0.01), supporting our hypothesis of lower levels of stress in these clones.

### Phenotypic and expression QTL linkages

We generated a new high-density genetic map (1.81 markers/10 kbp Additional file 3) from the Affymetrix S98 microarrays genotyping data for the 44 segregants. Linkage analysis was performed by the interval mapping method, for QTLs and eQTLs.

Three phenotypic QTLs were identified with a false discovery rate (FDR) threshold of 0.05: one QTL explaining flocculation on chromosome 1 encompassing *FLO1*, another explaining clumpiness and encompassing *AMN1* and a third, explaining fermentation rate (R70), on the left arm of the chromosome 16 (140 kbp), overlapping with the duplicated region and clearly triggered by the partial disomy. Limiting the population to the 31 segregants with no partial duplication of chromosome 16 eliminated the detection of the R70 QTL on chromosome 16. It also led to the detection of three QTLs for metabolites levels, mapping to chromosome 2 (208 kbp) for pyruvic acid and chromosome 14 (460 kbp) for succinic acid and glycerol.

Genome wide linkage analysis of gene expression led to the detection of 1063 eQTLs as summarize in Table 1. Dividing the FDR by two only slightly reduce the number of eQTLs, demonstrating the robustness of the data. A high amount (56%) of local-eQTLs (or cis-eQTLs, see Methods) were detected with higher average LOD than trans-eQTLs (Table 1). Some of the genes differentially expressed between the parents displaying cis-eQTLs corresponded to genes absent from one of the parental strains. As expected, genes from the three new regions acquired by horizontal transfer in EC1118 [13] and conserved in 59A formed three clusters (Additional file 2), each controlled in cis-regulations. We did not consider these genes, nor the 111 genes missing from EC1118 [13] for subsequent analyses, reducing the number of eQTLs to 967.

**Table 1:** Summary of eQTLs detected

**LODscore threshold**	**4**	**4.4**	**4 (without absent genes)**
trans-eQTL	468 (5.31*)	315	443
cis-eQTL	595 (8.78*)	508	524
Total	1063	823	967
FDR	0.10	0.05	0.10

* Average LODscore.

**Figure 3:**
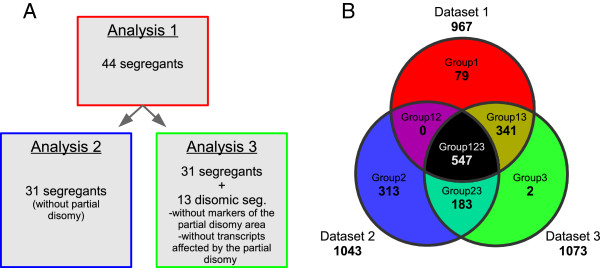
**Analyses to by-pass the effect of partial disomy on transcriptome. (A)** Principle of the analyses: Analysis 2 was the safest way to eliminate all the partial disomy effect and would have been applicable with a larger population. However, using a 31 segregants population reduced significantly our ability to detect small QTLs. Analysis 3 bypassed the majority of disomy effects while keeping a good detectability for the other traits. However, if the disomy has a partial effect on transcripts expression, this analysis kept this effect as a noise for eQTL detection. **(B)** Venn diagram for the comparison of the 3 analyses. The color used here will be used in Figure 4.

We found that 113 of the 1460 genes affected by the partial disomy displayed eQTLs, and 48 of these eQTLs mapped to the deleted or duplicated regions on chromosomes 8 and 16. Given that the partial disomy could affect the eQTL data, we adapted the analysis (analysis 1) to take this effect into account. Two new eQTL linkage analyses were performed as described in Figure 3A: (a) a search with a population limited to 31 non disomic segregants (analysis 2) (b) a filtering of the data for the 13 disomic segregants, eliminating the markers in the duplicated region and ignoring the 1460 genes affected by the partial disomy (analysis 3).

These two new data sets were compared with the first one on a Venn diagram Figure 3B and in Additional file 4. Most of the eQTLs (547) were common to all the three data sets (group 123 on Figure 3B) and corresponded to eQTLs with high LOD-score (cis-eQTLs). In analysis 2, restricted to the 31 non disomic segregants, the detection power was lower: at lod4, the FDR was 0.13. Data set 3 overlapped strongly with data set 1, with 888 of 967 eQTLs common to these two data sets. This suggests that the partial disomy slightly decreased our ability to detect true eQTLs, but had only a limited effect on the robustness of the data. The eQTL analysis was performed for the union of the three data sets (1465 eQTLs), taking the “Venn” group for each eQTL into account. We, therefore, concluded that we could overcome the effect of the partial disomy.

### Variations in expression networks are triggered by few loci resulting in the formation of hotspots

**Figure 4:**
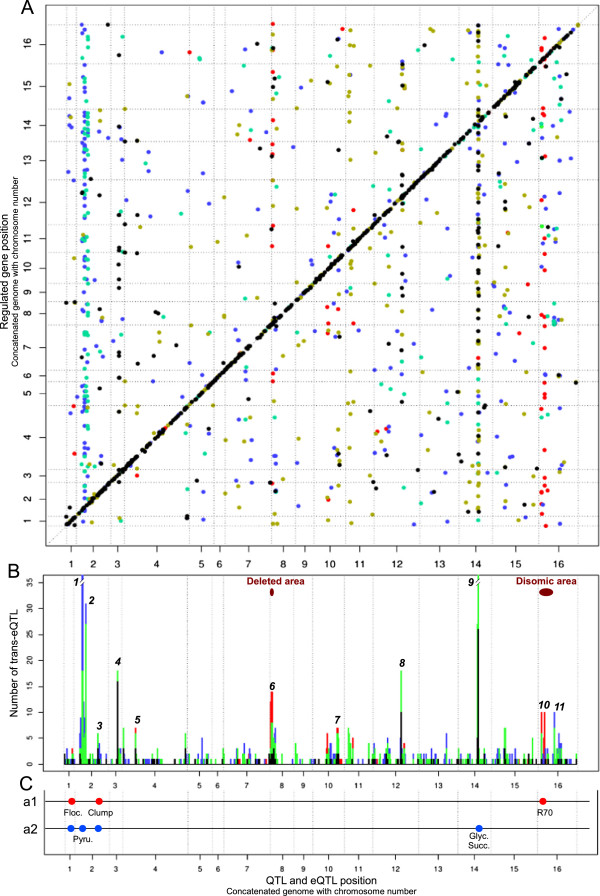
**QTL and eQTL localization. (A)** Localization of the 1465 eQTLs in function of the corresponding transcript position. Colors correspond to the group in the Venn diagram Figure 3B. Spot in the first diagonal correspond to cis-eQTLs. **(B)** Trans-eQTL density and formation of hotspot. Descriptions of each hotspot are in Table 1. Black peaks correspond to the trans eQTL in common of the 3 analyses, Green peaks correspond to trans-eQTL detected at least in analysis 3. Blue peaks correspond trans-eQTL specific of analysis 2. Red peaks correspond trans-eQTL specific of analysis 1. **(C)** Phenotypic QTLs detected with 44 segregants (a1, red) and with 31 segregants (a2, blue).

The locations of the phenotypic QTLs and eQTLs are summarized in Figure 4 and Table 2. Many eQTLs overlapped in several hotspots and most were common to the three analyses. However, the disomy was responsible for two hotspots on chromosomes 8 and 16 (hotspot 6 and hotspot 10, in red Figure 4B). Furthermore, 113 of the eQTLs discovered in the two new analyses are overlapping in two hotspots on chromosome 2, at 208 kbp and 283 kbp (hotspots 1 and 2 in blue/green Figure 4A-4B). Due to the genetic similarities between EC1118 and RM11-1a, some phenotypic QTLs and eQTLs are identical to those previously identified for the cross between BY4716 and RM11-1a (BxR population: hotspot 3, 4, 8 and 9, [25,34,35]).

**Table 2:** Hotspot description

**Hotspot**	**Localization**^**a**^	**Number of eQTL**^**b**^	**Functional enrichment**	**Phenotypic QTL**	**Knowon or candidate gene regulator**	**Functional validation**	**Comments**
1	chr2:207	66	No functional enrichment	Pyruvate	*SCT1*	Not realized	Hidden by the disomy, Sct1p is involved in lipid desaturation and diverges in the two strains by the size of a glutamic acid repeated sequence
2	chr2:277	47	No functional enrichment	-	Unknow	-	Hidden by the disomy
3	chr2:560	7	Daughter cell separation	Clumpness, OD, cell pop.	*AMN1*	Yvert et al. 2003 [34]	Impacting on cellular population estimation
4	chr3:198	20	Mating, pheromone	-	*MAT*	Brem et al. 2005 [35]	
5	chr4:309	9	Thiamine synthesis	-	*THI3*	Not realized	*THI3* bears two non-synonymous SNPs and a frame-shift in its coding sequence
6	chr8:20	27	Helicase	-	-	-	Disomy consequence
7	chr10:590	18	Mitochondrial protein	-	*GRR1*	Not realized	*GRR1* has a strong cis-eQTL (LOD = 13.4) and several alteration of its coding sequence
8	chr12:667	33	Fatty acid metabolism, detoxification	-	*HAP1/PDR8*	Yvert et al. 2003 [34]	Genes involved in the response to oxygen (nine targets of HAP1, Additional file 5)
9	chr14:456	101	Mitochondrial protein	Succinate, Glycerol	*MKT1*	Smith and Kruglyak 2008 [25]	Perturbation of the mitochondrial function affect the production of metabolites
10	chr16 left-arm	50	No functional enrichment	R70	-	-	Disomy consequence
11	chr16:370	17	chr8 translocated gene	-	*SSU1* translocation	Not realized	Translocation consequence

^a^Localization of the center of hotspot given in chromosome:kilo base pair.

^b^Number of total eQTL from the union of the 3 datasets.

However, we also found some linkages not previously identified. Hotspot 5 on chromosome 4 was found to be enriched in genes involved in thiamine biosynthesis pathway and is probably controlled by variations of the *THI3* regulator gene located in the hotspot area. Hotspot 7 on chromosome 10 brings together 18 genes with various functions (mitochondria, glucose sensing: *SNF3*, *YDL199c*, *GRR1*) and *GRR1* seams to be the most relevant candidate gene for the control of this hotspot. It is indeed involved in glucose repression and amino acid sensing [36].

**Figure 5:**
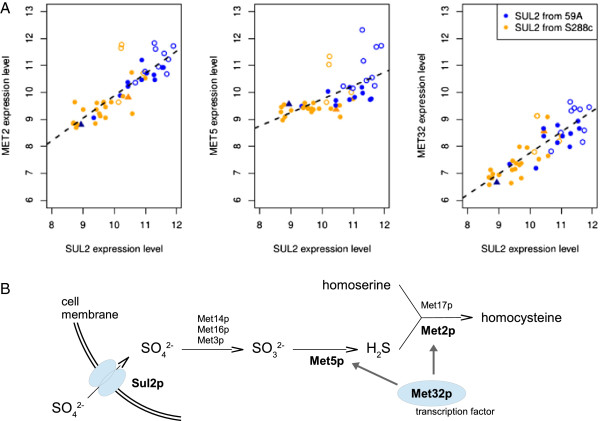
**Control of genes involved in sulfuric amino-acid synthesis by *SUL2 *expression. (A)** Correlation between *MET2, MET5* or *MET32* expression and *SUL2* expression. Orange spots correspond to the segregants with *SUL2* from S288c. Blue spots correspond to the segregants with *SUL2* from 59A. Open circles correspond to disomic segregants. Doted line corresponds to the linear regression. **(B)** Sulfate assimilation via *SUL2* and the four genes *MET2*, *MET5*, *MET32* and *MET3*. Genes in bold character display a linkage on *SUL2*.

We also found variations in several networks involving fewer genes but potentially involved in the wine-making process. The high-affinity sulfate permease gene, *SUL2*, has a cis-eQTL (LOD 4.5) overlapping with trans-eQTLs for three other genes of the sulfur pathway *MET2*, *MET5* and *MET32*. *SUL2* has two SNPs in its promoter (but none in the consensus sequences for transcription factors binding sites, Yeastract [37]) and five non-synonymous SNPs in its coding sequence. Changes in sulfate transport efficiency are clearly responsible for the higher expression level of the three other genes involved in sulfur amino-acid metabolism (Figure 5).

**Figure 6:**
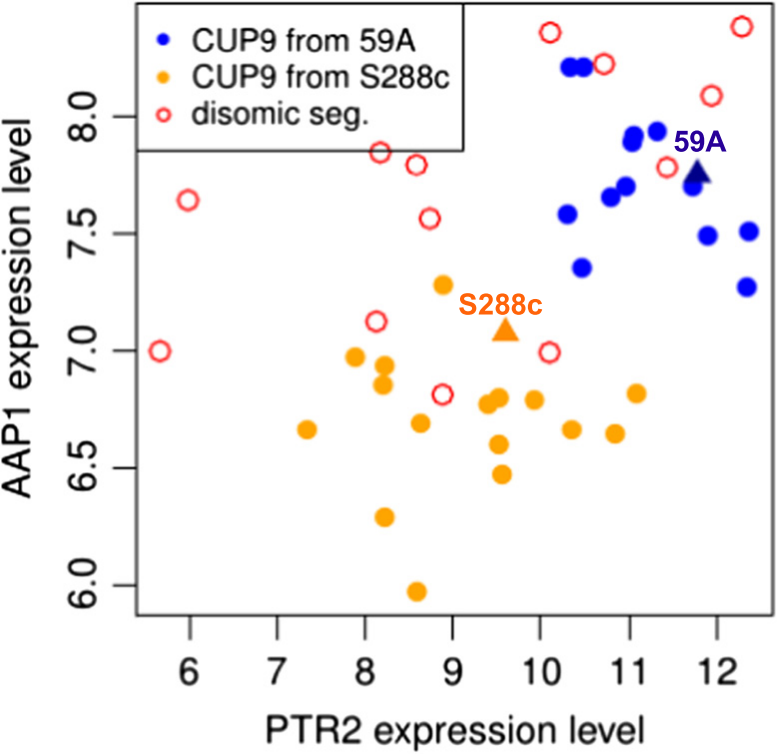
**Correlation between *PTR2 *and *AAP1 *expression.***CUP9* allelic origin is likely to control both genes. Red open circles correspond to disomic segregants, *CUP9* being localized in the left arm of chromosome 16. Orange spots correspond the segregants with *CUP9* from S288c. Blue spots correspond the segregants with *CUP9* from 59A.

Similarly, we observed a perturbation of the nitrogen uptake system that might have a significant impact on wine yeast performance [38]. The membrane peptide transporter gene, *PTR2*, and the arginine/alanine aminopeptidase gene, *AAP1*, both have eQTLs mapping to *CUP9* (chromosome 16) which encodes the known transcriptional repressor of *PTR2*. *CUP9* has two non-synonymous SNPs in its coding sequence and the 59A allele clearly increases the expression of *PTR2* and, probably, also that of *AAP1* (Figure 6). Byrd *et al.*[39] described the activation of *PTR2* by ubiquitin-dependent Cup9p degradation in the presence of peptide [40]. However, in the 59A strain, *PTR2* has a frame-shift in its sequence and is, therefore, probably non-functional [41]. The involvement of *CUP9* in the control of *AAP1* is consistent with a broader role of this regulator in peptide metabolism. The synthetic must used here did not contain peptides, but these molecules may have been generated by endogenous nitrogen metabolism. The mutation in 59A may act by modulating the repressive activity or degradation of Cup9p.

Finally, a flocculation QTL was found on chromosome 1, mapping to *FLO1* as previously described [24] (Figure 4C). Flocculation phenotype was found linked to *FLO1* expression level (Additional file 6). Surprisingly, multiple QTL searches on the basis of flocculation phenotype did yield a locus containing *FLO8*. However, a second QTL was found on chromosome 1, in a 45 kbp region (Additional file 6). This region contains the gene *OAF1* involved in fatty-acid and peroxisome biogenesis. It is possible that *OAF1* mutations triggered variation of cell surface hydrophobicity that could impact flocculation [42].

### Variations in detoxification mechanisms are triggered by mutations in transcription factor genes

In this linkage analysis, we detected changes in the detoxification network. Several membrane transporters involved in drug export displayed eQTLs (Table 3). Some of these genes were under local regulation (cis-eQTL): *SVS1*, required for resistance to vanadate, the two polyamine exporter, *TPO1* and *TPO2*, and the ion transporters *ALR2* and *SSU1*. The cis-eQTL of *SSU1* is due to the 8–16 translocation. Other detoxification system genes (e.g. *SNG1*, *PDR12*, *QDR2*) displayed distant control. Several eQTLs associated variations in the expression of these transporters with variations of zinc-finger transcription factor genes: *HAP1*, *PDR8, YRR1* and *WAR1*.

**Table 3:** Genes involved in detoxification having an eQTL

**Name**		**eQTL**				
**Standard**	**Systematic**	**Localization**^**a**^	**Lod score**	**Regulation**^**b**^	**Venn group**	**SGD Function**
**Ions Detoxification**						
*MNR2*	*YKL064W*	chr2:151	4,49	unknown	23	Putative magnesium transporter; has similarity to Alr1p and Alr2p which mediate influx of Mg2+ and other divalent cations
*ALR2*	*YFL050C*	chr6:30	5,88	**Self**	123	Probable Mg(2+) transporter; overexpression confers increased tolerance to Al(3+) and Ga(3+) ions; plays a role in regulating Ty1 transposition
*SSU1*	*YPL092W*	chr16:371	5,92	**Self**	123	Plasma membrane sulfite pump involved in sulfite metabolism and required for efficient sulfite efflux; major facilitator superfamily protein
**Drug Transport**						
*TPO2*	*YGR138C*	chr7:761	17,28	**Self**	123	Polyamine transport protein specific for spermine; localizes to the plasma membrane; transcription of TPO2 is regulated by Haa1p; member of the major facilitator superfamily
*SNG1*	*YGR197C*	chr15:639	5,24	*YRR1*	1	Protein involved in nitrosoguanidine (MNNG) resistance; expression is regulated by transcription factors involved in multidrug resistance
*ERC1*	*YHR032W*	chr2:624	4,09	unknown	13	Member of the multi-drug and toxin extrusion (MATE) family of the multidrug/oligosaccharidyl-lipid/polysaccharide (MOP) exporter superfamily
*PDR11*	*YIL013C*	chr12:659	4,78	*HAP1/PDR8*	123	ATP-binding cassette (ABC) transporter multidrug transporter involved in MDR; mediates sterol uptake when sterol biosynthesis is compromisedregulated by Pdr1p
*QDR2*	*YIL121W*	chr12:680	10,31	*HAP1/PDR8*	123	Multidrug transporter of the major facilitator superfamily required for resistance to quinidine barban cisplatin and bleomycin; may have a role in potassium uptake
*GEX2*	*YKR106W*	chr11:658	13,66	**Self**	123	Proton:glutathione antiporter localized to the vacuolar and plasma membranes; almost identical to paralog Gex1p; potential role in resistance to oxidative stress
*TPO1*	*YLL028W*	chr12:86	9,89	**Self**	123	Polyamine transporter that recognizes spermine putrescine and spermidine; catalyzes uptake of polyamines at alkaline pH and excretion at acidic pH
*TPO4*	*YOR273C*	chr12:659	4,61	*HAP1/PDR8*	13	Polyamine transport protein recognizes spermine putrescine and spermidine; localizes to the plasma membrane; member of the major facilitator superfamily
*PDR12*	*YPL058C*	chr13:116	7,50	*WAR1*	123	Plasma membrane ATP-binding cassette (ABC) transporter weak-acid-inducible multidrug transporter required for weak organic acid resistance; regulated by War1p
**Transcription factor**						
*HAP1*	*YLR256W*	chr12:673	6,22	**Self**	123	Zinc finger transcription factor involved in the complex regulation of gene expression in response to levels of heme and oxygen
*PDR8*	*YLR266C*	chr16:138	5,00	unknown	2	Transcription factor; targets include ATP-binding cassette (ABC) transporters, major facilitator superfamily transporters, and other genes involved in the PDR phenomenon
*WAR1*	*YML076C*	chr12:673	6,57	*HAP1/PDR8*	123	Homodimeric Zn2Cys6 zinc finger transcription factor; binds to a weak acid response element to induce transcription of PDR12 and FUN34
*YRR1*	*YOR162C*	chr15:639	5,03	**Self**	13	Zn2-Cys6 zinc-finger transcription factor that activates genes involved in multidrug resistance; paralog of Yrm1p acting on an overlapping set of target genes
*FZF1*	*YGL254W*	chr7:21	4,02	**Self**	123	Transcription factor involved in sulfite metabolism, identified regulatory target is SSU1, overexpression suppresses sulfite-sensitivity
**Other/Unknown**						
*SVS1*	*YPL163C*	chr16:251	9,55	**Self**	123	Cell wall and vacuolar protein required for wild-type resistance to vanadate
*YKR104W*	*YKR104W*	chr11:651	4,63	**Self**	13	Putative transporter of the multidrug resistance-associated protein (MRP) subfamily; contains a stop codon in S288C
*YLR046C*	*YLR046C*	chr12:238	5,72	**Self**	123	Putative membrane protein transcription is activated by paralogous transcription factors Yrm1p and Yrr1p along with genes involved in multidrug resistance
*YJL216C*	*YJL216C*	chr10:42	11,36	**Self**	123	Protein of unknown function similar to alpha-D-glucosidases; transcriptionally activated by both Pdr8p and Yrm1p along with other genes involved in the PDR phenomenon
*YLR179C*	*YLR179C*	chr12:689	4,08	*HAP1/PDR8*	23	Protein of unknown function transcription is activated by paralogous proteins Yrm1p and Yrr1p along with proteins involved in multidrug resistance; not essential

^a^Localization of the center of hotspot given in chromosome:kilo base pair.

^b^“**Self**” mean the eQTL is closer than 40 kpb to the gene controlled.

**Figure 7:**
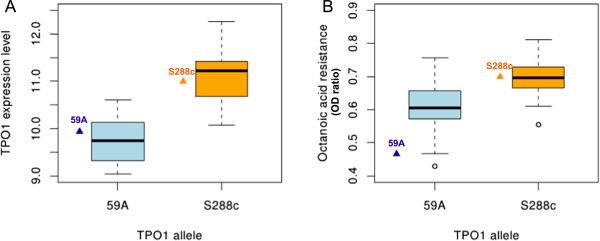
***TPO1 *allele controls is own expression and octanoic acid resistance. (A)** Box plot showing *TPO1* allele effect over *TPO1* expression level in the population. **(B)** Box plot comparing segregants resistance to octanoic acid by *TPO1* allele (t-test Pv<0.005). Values for parental strains are indicated.

The *TPO1* gene, which displays a cis-eQTL, has three SNPs in its promoter area and three non synonymous SNPs in its coding sequence. The S288c allele of *TPO1* is more strongly expressed than the wine yeast allele. As *TPO1* is involved in octanoic acid resistance [3], we investigated the effect of variations of *TPO1* expression on octanoic acid resistance. We measured population growth in the synthetic must medium (pH3.3) supplemented with octanoic acid (0.2 mM). Octanoic acid resistance dependent principally on the *TPO1* allele (*t*-test Pv<0.005, Figure 7) but was also weakly correlated with *TPO1* expression level (data not shown). Our data therefore suggest that the form of *TPO1* encoded by the S288c allele is more effective, conferring a higher octanoic acid resistance.

*PDR11*, *TPO4* and *QDR2* all had an eQTL in hotspot 8 mapping to *HAP1* (Table 3). Another gene, *PDR8*, encoding a pleiotropic drug resistance transcription factor lies in the same locus, 25 kbp away from *HAP1*, and may control some of these genes (Additional file 5). Consistent with this hypothesis, Steyer *et al.*[32], recently showed that high nerolidol production were associated with the S288c allele of *PDR8*. They demonstrated that *QDR2* regulation by *PDR8* accounted the variation of nerolidol release into the medium. We assessed the impact of *PDR8* allelic variation on gene expression by performing an allelic switch for *PDR8* and constructed 59A strain expressing the *PDR8* allele from S288c (59A *PDR8*-S288c). We compared the transcriptome of this strain with that of wild type 59A in fermentation conditions (the same conditions as for the global analysis). We detected variation of expression for very few genes (adjPv<0.01, Table 4). *QDR2* was the gene most strongly upregulated by allelic replacement, with a LogFC of 2.24, consistent with the results of Steyer *et al.*[32]. Among the other targets of *PDR8*, we only found *YLR179c* controlled by the allelic form of *PDR8*. Both *QDR2* and *YLR179c* had an eQTL mapping to *PDR8*. In addition to a slight down regulation of *PDR8* itself, we identified three other genes (*YLR149c*, *GIT1* and *QDR1*) as upregulated in the constructed strain and potentially playing a role in detoxification. These genes have not previously been described as *PDR8*-controlled. We did not detect the targets of *PDR8* reported by Hikkel *et al.*[43]. This was probably due to the specific conditions of alcoholic stress and nutrient starvation used in our study. Furthermore, we performed an allelic switch with a smaller impact than the over-expression of *PDR8* used in other studies. Finally, only *QDR2* and *YLR179c* were controlled by *PDR8* in hotspot 8, suggesting that expressions of the other genes are triggered by *HAP1*.

**Table 4:** **Gene expression modified by *PDR8 *** **allelic switch**

**ORF**	**Name**	**eQTL**	**LogFC**	**AdjPv**	**SGD function**
*YIL121W*	*QDR2*	hotspot8	2,234	<10e-05	Multidrug transporter of the major facilitator superfamily required for resistance to quinidine barban cisplatin and bleomycin
*YLR179C*	*YLR179C*	hotspot8	0,897	0,0026	Protein of unknown function transcription is activated by paralogous proteins Yrm1p and Yrr1p along with proteins involved in multidrug resistance
*YLR149C*	*YLR149C*	-	1,349	<10e-05	Putative protein of unknown function; overexpression causes a cell cycle delay or arrest; null mutation results in a decrease in plasma membrane electron transport
*YCR098C*	*GIT1*	-	0,548	0,005	Plasma membrane permease, mediates uptake of glycerophosphoinositol and glycerophosphocholine as sources of the nutrients inositol and phosphate
*YIL120W*	*QDR1*	-	0,472	0,0046	Multidrug transporter of the major facilitator superfamily, required for resistance to quinidine, ketoconazole, fluconazole, and barban
*YLR266C*	*PDR8*	chr16:138	−0,493	0,0032	Transcription factor; targets include ATP-binding cassette (ABC) transporters, major facilitator superfamily transporters, and other genes involved in the pleiotropic drug resistance (PDR) phenomenon

**Figure 8:**
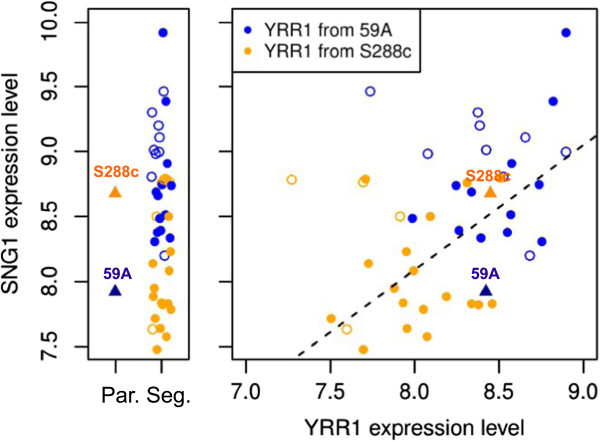
***YRR1 *control over *SNG1 *expression.** Orange spots correspond the segregants with *YRR1* from S288c, blue spots correspond the segregants with *YRR1* from 59A. Open circles correspond to disomic segregants, There is a permutation between parent and the allelic effect in the segregants population, involving polygenic control.

The gene paralog of *PDR8*, *YRR1*, displays a cis-eQTL on chromosome 15 and has four SNPs in its promoter region and six non-synonymous SNPs in its coding sequence. *SNG1*, one of the targets of *YRR1* responsible for nitrosoguanidine resistance [44], has an eQTL mapping to the position of *YRR1*. Surprisingly, we found no linkages of this locus to other *YRR1* target genes. Moreover, the inverse patterns of parental behavior and locus segregation for *SNG1* expression indicates that other loci are involved in *SNG1* control (Figure 8).

**Figure 9:**
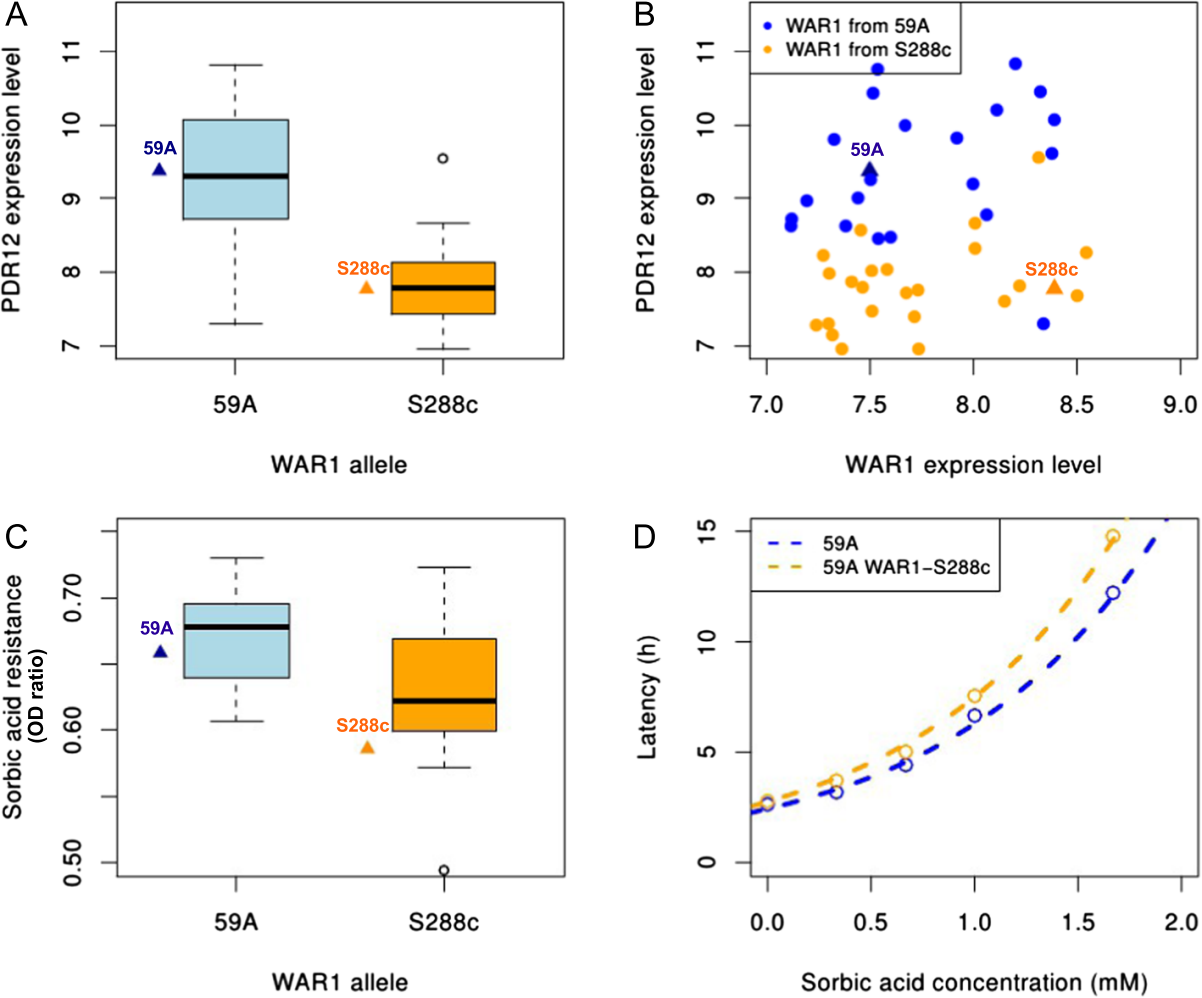
**Control of sorbic acid resistance by *WAR1 *allele. (A)** Box plot showing *WAR1* allele effect over *PDR12* expression level in the population. Values for parental strains are indicated. **(B)** Confrontation of *WAR1* expression level and *PTR12* expression level show no correlation. Orange spots correspond the segregants with *WAR1* from S288c. Blue spots correspond the segregants with *WAR1* from 59A. **(C)** Box plot comparing segregants resistance to sorbic acid by they *WAR1* allele (t-test Pv<0.005). Values for parental strains are indicated. **(D)** Lag phase duration in function of the sorbic acid concentration for 59A wildtype and for the allelic replacement of *WAR1*.

The *PDR12* gene, encoding a plasma membrane ABC transporter responsible for organic acid efflux, had an eQTL mapping to its known transcription factor *WAR1* (chromosome 13), an activator of weak acid stress response. The *WAR1* allele of 59A has five non synonymous SNPs in its coding sequence and was associated with higher levels of *PDR12* expression level (Figure 9A). In addition, *WAR1* was itself one of the genes for which expression was affected by *HAP1* variation (hotspot 8). The level of *WAR1* expression had no significant impact on *PDR12* transcript abundance. Instead *PDR12* expression levels were influenced by the type of *WAR1* allele (Figure 9B). *PDR12* was the only known *WAR1-*dependent gene with an eQTL mapping at *WAR1* (even with a LODscore below the threshold) [45]. As *PDR12* has been implicated in sorbic acid resistance [46], we investigated whether variation of the expression of this gene modulated resistance to this acid. We assessed the growth of segregants in the synthetic medium (pH3.3) supplemented with sorbic acid (0.5 mM, see Methods). Sorbic acid resistance appeared to be partly controlled by the *WAR1* allele (Figure 9C, *t*-test Pv<0.005) consistent with the effect of *WAR1* allele on *PDR12* expression. We then performed an allelic switch for *WAR1* and constructed a 59A strain expressing the *WAR1* allele from S288c (59A *WAR1*-S288c). The growth profiles of the strains were monitored in the synthetic must medium supplemented with various amount of sorbic acid. The lag phase was longer for 59A *WAR1*-S288c than for 59A wild-type (Figure 9D). This phenotypic difference was subtler than that observed with a knockout strain, but these results nevertheless confirm that sorbic acid resistance is linked to *PDR12* expression and to *WAR1* genetic variation. Variations of detoxification networks may thus be linked to variations of several transcription factor genes. Two of these linkages were validated by allelic switch experiments. Moreover, we demonstrated that the levels of resistance to octanoic acid and sorbic acid were clearly modulated by these genetic variations.

As polymorphism may result from specific adaptation, we tried to infer the phylogeny of War1p and Yrr1p from nucleotidic sequence available for different strains in SGD. Interestingly; the two allelic versions (wine and laboratory) of these two genes diverge from their *S. paradoxus* orthologue, and are clearly divergent but apparently in a different manner. For *WAR1*, S288C and 59A alleles are located on different branches, whereas for *YRR1*, the phylogeny indicates two clusters one containing palm wine and Asian alleles and the second cluster containing 59A wine, rum and bread alleles, as well as S288C alleles. However in this case S288C and 59A are also located on different branches of this cluster (Additional file 7, MEGA 5 [47]). In order to detect if these two genes have evolved neutrally, we performed a McDonald Kreitman test calculating the neutrality index (NI) [48]. This test was applied to a set of 15 *WAR1* alleles from strains isolated from various substrates and NI was 2.28 (Pv = 0.02), indicating a significant excess of non-neutral mutations. As the cluster of Asian alleles might inflate this test, we removed the sequence and performed the test again, which indicate a similar result (NI = 2.56 and Pv = 0.03). We performed the similar analysis on *YRR1* alleles. We obtained NI = 2.51 (Pv = 0.001) for the whole set of alleles and NI = 3.93 (Pv = 0.002) after removal of the Asian and palm wine alleles. This suggests that *WAR1* and *YRR1* do not evolve neutrally: these two genes are either subject to the accumulation of slightly deleterious mutations that are eliminated by negative selection during speciation, or alternatively they present substantial diversity that might be associated with balanced selection resulting from specific adaptation of the strain to wine fermentation conditions.

## Discussion

In this study, we developed a genetical genomic approach to get insight into variation of gene regulatory networks during alcoholic fermentation and to assess the impact of this variation on wine yeast properties. We mapped 1465 eQTLs with 601 local eQTLs and 864 distant eQTLs (several distributed in 11 hotspots). As heritability exceeded 80% for 80% of the transcripts, genetic variations clearly had a major impact on global mRNA levels. The differences between the transcripts differentially expressed between the two parental strains and those displaying eQTLs confirmed that multigenetic controls are underlying most variations in transcripts abundance. These observations are consistent with the results obtained for other genetical genomic approaches in various organisms [30]. We observed several overlaps between phenotypic QTLs (glycerol, pyruvate) and eQTLs, consistent with the involvement of regulatory networks variation in yeast trait variations. Some of these linkages have been described before (clumpiness, floculation, mating type), whereas others are probably specific to our conditions or strains. We show that various mechanisms contribute to variations of expression and highlight the role of transcription factors for toxic compound export and the contribution of large chromosomal variations, a partial chromosomal duplication in this case, to the control of fermentation rate.

The transcriptome analysis was performed during the stationary phase in starvation and alcohol stress. Therefore our approach was adapted to describe regulatory change associated to this condition. However, variations in regulatory network involved in nitrogen consumption and cell growth were not considered here. For example, expressions of only few genes were found correlated to Rmax.

### Genetic control of fermentation rate

Ambroset *et al.*[4] showed that fermentation rate (Rmax) depended on the PABA biosynthesis capacity and *ABZ1* allele. By bypassing the effect of *ABZ1*, we identified another genetic control of the fermentation rate: the improvement of fermentation kinetics by partial disomy of the left arm of chromosome 16. In the system studied here, this partial disomy was present only in the offspring, due to cross carried out. This link cannot therefore be considered a “real” QTL, but it nevertheless highlights the role of gene copy number variations in the control of a key phenotype [11].

The mechanisms underlying the improvement of fermentation rate associated with this disomy are unclear, however, and will not be easy to decipher, given the size of the chromosomal region and the large number of genes it contains (190 genes). However, candidate genes may be identified on the basis of their patterns of expression, because the fermentation phenotype is thought to be triggered by a change in expression level. One or several of the 155 genes overexpressed in the translocated area may improve tolerance to starvation or, potentially, to alcohol. We investigated the properties of the set of genes in this region of chromosome 16 and evaluated their correlation with fermentation rate (R70). We observed that levels of expression of *SAM3* and *SAM4*, involved in S-adenosyl methionine (SAdM) pool control, were correlated with R70. Interestingly, the expression levels of other genes, not located in the duplicated region, involved in SAdM synthesis (*SAM1*, *SAM2*) and involved in methyl transfers (*CHO2*, *NNT1*, *SET7*) were also correlated with R70. We suspect that *SAM3* and *SAM4* improve of the fermentation rate of the disomic segregants by increasing the availability of SAdM. However, the addition of SAdM (final concentration: 0.1 μM) to the fermentation medium did not improve the fermentation rate of the 59A strain (data not shown). We previously showed that the methyl donor synthesis and methionine biosynthesis pathways had a strong impact on fermentation rate [4], highlighting the key role of these metabolic pathways in alcoholic fermentation. Additional experiments are required to determine the mechanisms underlying this control of fermentation rate.

**Figure 10:**
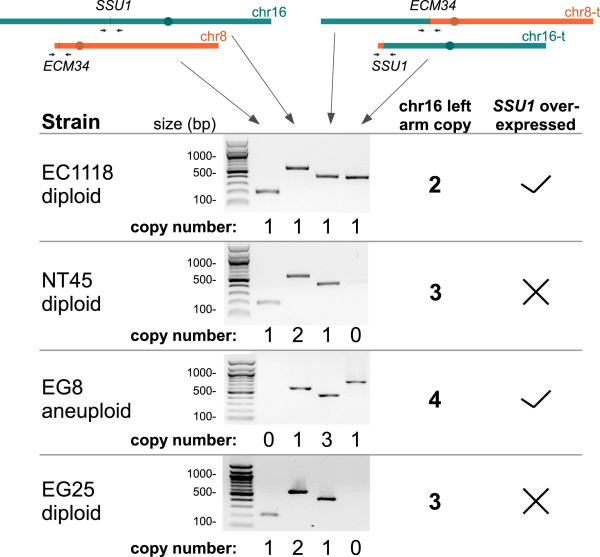
**Confirmation by PCR of chromosome left arm amplification.** Confirmation over three strain: NT45, EG8 and EG25 selected by aCGH data. EG8 displays a chromosome 8 trisomy. *SSU1* over-expression is carried by chromosome 16 translocated.

As this chromosomal amplification increased the fermentation capacity of wine yeasts, we checked for its presence in wild and industrial strain. Dunn *et al.*[12] reported a aCGH analysis of 83 *S. cerevisiae* strains including 69 wine strains. We used their data to search for a partial trisomy (for diploid strains) of the left arm of chromosome 16. We detected such partial trisomy in the wine yeast strain NT45. Moreover, in their characterization of the *S. cerevisiae/S. kudriavzevii* triploid hybrid EG8, Erny *et al.*[49] detected a major amplification (four copies) of the left arm of the *S. cerevisiae* chromosome 16. The diploid *S. cerevisiae* strain EG25, which is genetically related to the *S. cerevisiae* moiety of the EG8 genome (with no *S. kudriavzevii* DNA), also displays trisomy of this region (Legras JL, personal communication). We determined the form of the chromosomes 16 and 8 (normal or translocated) present, by PCR for these three strains (Figure 10). The NT45 and EG25 strains carry three chromosomes 16 left arms, originating from two normal chromosomes 16 and one copy of chromosome 8 carrying the translocation from chromosome 16. The hybrid aneuploid strain EG8 carries four left arms of chromosome 16: one from a normal chromosome 16 and three from the three chromosomes 8, all carrying the translocation. This findings confirm that amplification of the left arm of chromosome 16 occurs in wild/industrial strains. The situation described here suggests a spectacular evolutionary event occurring in two independent steps: a translocation selected due to its effect on sulfite resistance followed by amplification of the chromosomal region concerned, improving fermentation properties.

We evaluated the contribution of the disomy effect to eQTL linkage, by modifying the input dataset. We found that a large set of new eQTLs had been masked by the effect of this disomic. Most of these eQTLs (113) formed 2 hotspots on chromosome 2. Several of the genes controlled by these hotspots were found correlated with R70. Thus, these loci may partially control fermentation rate, despite the lack of detection of a QTL for this phenotype.

### Importance of variations in detoxification systems

**Figure 11:**
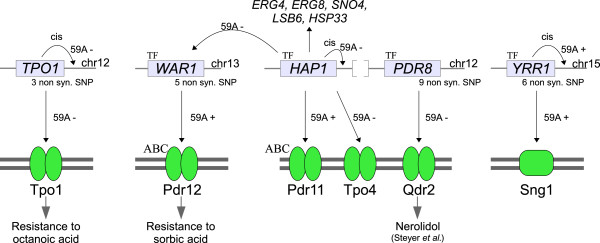
**Summary of the variations in the detoxification regulatory network.** Small arrows represent an allelic control on expressions. ABC transporter and transcription factor (TF) are indicated.

The analysis of functional enrichment for all eQTLs (in FunSpec [33]) highlighted a set of 22 eQTLs linking genetic variation to expression level of genes involved in detoxification. Our data show the importance of variations in transcription factor genes and their impact on detoxification systems: *PDR8*, *WAR1* and *YRR1*, in addition to *HAP1* (as summarized in Figure 11).

During fermentations, yeast produces toxic compounds that may lead to stuck fermentations [50]. This is the case for the medium-chain fatty acids generated as by-products of lipid biosynthesis. *PDR12* has been shown to be involved in weak organic acid [46], and Legras *et al.*[3] have shown that *PDR12*, together with *TPO1*, is also a key transporter involved in octanoic acid and decanoic acid resistance. We were able to link genetic variations of *TPO1* to its expression and to octanoic acid resistance. *PDR12* has an eQTL in the gene encoding its known transcription factor, *WAR1*, that we could link to variations of sorbic acid resistance, but not to octanoic acid resistance, suggesting that the allelic effect of *TPO1* outweighs the control of medium-chain fatty acid resistance.

For the same population, Steyer et al. [32] shown that the allele of *PDR8* gene controls the export of nerolidol via *QDR2*. We confirm here that *PDR8* allelic variation controls the expression of *QDR2* and *YLR179c*, the function of which remain unknown. We observed that several drug exporters (*TPO1*, *TPO2*, *TPO4* and *QDR2*) were less strongly expressed by the wine form, consistent with the deletions in wine yeasts reported by Dunn *et al.* (*PDR3*, *SNQ1*, *QDR1*[11]). The exact physiological role of several of these transporters remains unclear. Qdr2p was initially identified as a drug efflux system but its natural targets remain a matter of debate [51,52]. It was subsequently suggested that Qdr2p might export amino acids in starvation conditions [53]. Such a function would be consistent with the lower levels of expression of this gene in wine yeast, given the major effect of nitrogen homeostasis on wine yeasts fermentation capacity.

In addition to the toxic compounds produced by the yeast during fermentation, natural grape musts contain various phenolic compounds [54], which act as key wine preservatives and contribute to aging. Stilbene compounds are clearly toxic to fungi, such as *S. cerevisiae* and *Aspergillus flavus*[55]. Pan *et al.*[56] studied the transcriptomic response to pterostilbene (an analog of resveratrol) and show that *YRR1* and *SNG1* were among the upregulated genes in response to the drug. We identified eQTLs for these two genes, which were upregulated by the wine allele of *YRR1* transcription factor encoding gene.

As the polymorphism observed here may result from specific adaptation to exposure to these compounds, we tried to infer to phylogeny of War1p and Yrr1p and evaluate if these genes evolve neutrally. Interestingly the McDonald Kreitman test [48] revealed a non neutral evolution for these two genes (NI calculated was greater than two): this indicates very likely that the substantial diversity observed for *WAR1* and *YRR1* results from balanced selection due to specific adaptation of the strain to wine fermentation conditions. This is consistent with the analysis of Steyer *et al.*[32], who reported also a non neutral evolution of *PDR8* according to the results of a McDonald test.

Unlike Yvert *et al.*[34], who reported that distant QTLs were not often due to variations of transcription factor genes, we found that most of the variation of detoxification gene expression was linked to transcription factors. Surprisingly, variations in these three genes (*PDR8*, *YRR1*, *WAR1*) encoding zinc-finger transcription factors modified the expression of only one or two of their known targets. This may be partly due to the different environment in which *PDR8* targets have been characterized. We performed our expression analysis in stationary phase under a stressful condition whereas cell growths in rich medium were used for the Chip on Chip assay of Hikkel *et al.*[43]. In addition, Jothi *et al.*[57] have shown that these transcription factors belong to the lower layer of the hierarchy of the yeast transcription factors network, with direct effects on a limited number of targets. This may make them more tolerant to variation than genes higher up in this hierarchy.

### Regulatory network variations involved in wine fermentation

In this study, we investigated, for the first time, the expression profiles of the genes present in the three regions introgressed into EC1118 by horizontal transfer [13]. As we worked with a second parental strain devoid of these three regions, we could not identify real eQTLs for these genes. We searched for links, using the populations of segregant possessing each region (half of segregants). On chromosome 6, at 145kpb, an eQTL was identified that trigger the expression of the two genes encoding polypeptide transporters (FOT) describes by Damon *et al.*[41]. The new fructose transporter gene, *FSY1*, [58] also had an eQTL on chromosome 13, at 446 kbp (data not shown). No clear candidate gene was identified in these loci, but our findings could lead to revealing the role of these new genes in fermentation. No phenotypic QTL or trans-eQTL were found in these three regions. More detailed analysis, with a larger set of segregants, should be carried out to assess the variations of expression associated with these regions.

The phenotypic data indicated that most of the traits considered were under multigenic control. This may reflect the large genetic distance between our two parental strains, but Marullo *et al.*[59] aslo found, in a study using two commercial wine yeasts and a population of 51 segregants, a preponderance of multigenic traits, such as ethanol tolerance and kinetic properties. We were nevertheless able to associate several phenotypic QTLs with eQTLs. Pyruvic acid levels, which were strongly correlated with R70, were linked to a major hotspot on chromosome 2. A candidate gene, *SCT1*, was identified. This gene is involved in lipid desaturation, suggesting that it may act by modulating the plasma membrane stability under alcoholic stress.

However, within the eQTLs set, several new linkages are probably involved in the management of the multi-stress conditions in wine fermentation. Changes were detected for genes involved in glucose sensing (*GRR1* cis-eQTL that may control *SNF3* expression) or thiamine synthesis (affected by *THI3*). Functional validations of these results are required, together with further investigations to determine the role of such genetic variation in the adaptation of strains to the wine making process. We also showed that the sulfur amino-acid pathway (*MET32*, *MET2*, *MET5*) was partially enhanced by *SUL2* mutation, probably through effects on sulfate uptake. Such activation has not been described before and may reflect the conditions of nitrogen starvation in this study.

## Conclusion

In this study, we developed an approach to deciphering the variation of transcriptional networks in yeast, during an industrial wine-making process resulting in multiple stresses. The results obtained were consistent with those of other studies. However, the wine fermentation conditions used here led to the identification of specific variations of regulatory networks not revealed previously in classical laboratory environment. We show that expression variations affect networks of genes involved in various pathways and cellular functions, through diverse mechanisms involving both local and distal regulations and copy number changes driven by chromosomal rearrangements. We focus here on variations of detoxification systems and identified and we confirm the key role of variations in transcription factor genes in modulating the expression of these systems. These results also raise questions about the role of these export systems in fermentation conditions, in which cells are starved and stressed.

The eQTL linkage analysis was generally highly consistent, but most of the phenotype changes were not linked genetic variations and were considered to be under multi-locus control. We found that fermentation rate depended on a gene copy numbers effect of a duplication of the left arm of chromosome 16. This partial disomy was also found in natural yeasts and appears to be a potential new target for yeast optimization for the wine making process.

## Methods

### Strains, growth conditions and fermentation medium

The two parental strains used for the segregating population are S288c (*MAT*α; *SUC2*, *gal2*) strain and 59A (*MAT*a; ho), which is a haploid derivative of EC1118 (HO/ho), an industrial wine strain [4]. Mutations between the two parental strains are accessible in the GenYeasTrait database previously described (http://genome.jouy.inra.fr/genyeastrait/[4]).

We increased the population of 30 F1 segregants described in [4] with 14 randomly selected segregants. Thereby we disposed of two complete tetrads in the analyzed population, and the other segregants were selected from 24 different asci of two or three viable spores. The phenotyping was done on fermentation in 0,9l of synthetic SM425 medium (with 425 mg/l of assimilate nitrogen pH3.3 and previously describe as SM300 [60]) which mimics natural grape must. This medium was supplemented by 1 mg/l of p-aminobenzoic acid (PABA, sigma A-9878), 14.5 mg/l of iron (III) chloride (FeCl_3_, sigma F-7134), 10 μl/l of antifoam silicone (rhodorsil Prolabo 27429.297). The sugar source was glucose (10%) and fructose (10%) and the quantity of anaerobic factor was reduced by ½. After a reactivation of strain on YPED (48 h) and a preculture in shake flasks containing 30 ml of SM425 (12 h), 900 ml of medium were inoculated at 10^6^ cell/ml. Fermenters were equipped with airlock to maintain anaerobiosis. Fermentation were performed at 24°C and preculture at 28°C.

### Fermentation kinetics and phenotypic measures

Each parental fermentation and phenotypic measures were realized in four replicates and segregants fermentation in two replicates. The fermentation kinetics were measured by the loss of weight due to CO_2_ release. The weight was record every 20 min and the data were smoothed by polynomial method. The final population size was estimated at 45 g/l of CO_2_ release and 66 gCO_2_/l using electronic particle counter (Beckman Coulter) and optic density at 600 nm (OD_600_, spectrophotometer SECOMAN-UVLine9400). Dry weights were measured at 88 gCO_2_/l by filtration using nitrocellulose filters (pore size 0.45 μm; Millipore) and drying at 100°C (48 h).

The metabolic compounds amounts in the medium was measured after the end of the fermentation by high-pressure liquid chromatography (HPLC) using ion-exclusion column (HPX-87H BIO-RAD) with 8 mM H_2_SO_4_ mobile phase. Glucose, ethanol, glycerol and succinate were detected by refractometry. Acetic acid and pyruvic acid were detected by UV absorption (as described in [61]). Measures repeatability between replicate was controlled and the phenotype heritability *H*^*2*^ was calculated as follow (describe in [35]):

$$H^{2}=\frac{\mathrm{Va}r_{\mathrm{seg}}-\mathrm{Va}r_{\mathrm{env}}}{\mathrm{Va}r_{\mathrm{seg}}}\times 100$$

where *Var*_*env*_ is the pooled variance among parental measurement and *Var*_*seg*_ is the variance among phenotype values for the segregants. Phenotypes with low *H*^*2*^ are not statistically valid and were discarded.

Resistance phenotyping was performed in the same SM425 medium (pH3.3) than fermentation with a completion of the right amount of toxic compound stock solution and ethanol for a final rate of 1.7% (vol/vol). Octanoic acid stock solution: 69.3 mM in ethanol. Sorbic acid stock solution: 33.7 mM in water. The growth was taken with OD_600_ with and without toxic compound, and resistance indicator was measured by latency differences, ratio of specific growth rate and ratio of OD_600_ at 20 hours (end of exponential stage). We here only considerate the ratio of OD_600_, showing the lower experimental variation. Three replicates were done for each parental growth.

### Molecular biology and strains construction

*PDR8* allelic switch in 59A was obtained in three steps: 1) depletion of *PDR8* in 59A using hphMX4 cassette for hygromycin resistance (pAG32). Primers sequences for cassette amplification and verification were obtained from Euroscarf. 2) preparation of a replacement cassette containing *PDR8*-loxP-kanMX4-loxP by the insertion of loxP-kanMX4-loxP (pUG6 [62]) in the terminator of *PDR8* in S288c strain (primers in Additional file 8). 3) allelic switch by depletion of the hphMX4 cassette of 59A *PDR8Δ::hph* by the *PDR8*-loxP-kanMX4-loxP replacement cassette from S288c and selection on YEPD containing G418 (200 μg/l). The loss of hphMX4 cassette was controlled by PCR and the absence of growth on hygromycin (200 μg/l).

*WAR1* allelic switch in 59A was obtained in two steps: 1) depletion of *WAR1* in 59A using loxP-kanMX4-loxP (pUG6). Primers sequences for cassette amplification and verification were obtained form Euroscarf. 2) allelic switch by depletion of the kanMX cassette by *WAR1* sequence form S288c DNA (primers in Additional file 8) and selection on YEPD containing 300mg/l sorbic acid, pH4.2. The loss of kanMX cassette was controlled by PCR and the absence of growth on YEPD supplemented by G418 (200 μg/l).

The 8–16 translocation was confirmed by PCR, using the same primers than Pérez-Ortín *et al.*[10].

### Transcriptomic profiling

The transcriptome profiling was performed only one time for each segregants and in three technical replicates for each two biological replicates of the parental strains. At 66 gCO_2_/l release (70% of fermentation progress), 10^9^ cells were sampled, pelleted, washed with DEPC-treated water and freezed in methanol at −80°C. This condition is corresponding to late stationary phase; cells are not in transition situation which could have increase experimental variation on transcriptome. Total RNA extractions were performed with Trizol reagent (Gibco BRL, Life Technologies), purified by isopropanol precipitation then with RNeasy kit (Qiagen). Cy3-labeled cRNA was synthesized with the One color RNA Spike-In kit (Agilent Technologies) and purified with RNeasy kit (Qiagen). Quality and quantity of RNA were controlled at each step by spectrometry (NanoDrop 1000, Thermo Scientific).

Agilent gene expression microarrays 8x15k was used for the micro array hybridization, with one-color method (Agilent Technologies, Santa Clara, CA, USA). Array design is based on ID 016322 completed with the 39 genes from the new region of EC1118 [13] and available on GEO with GPL16012 as accession number. A quantity of 600ng of labeled cRNA were hybridized for 17 h in 65°C in a rotative hybridization oven (Corning) using the Expression Hybridization kit (Agilent Technologies, 5188–5242). Plates were washed with expression wash buffer kit (Agilent Technologies, 5188–5325 5188–5326). The array pictures were analyzed on a GenePix 4000B laser Scanner (Axon Instruments) and with the GenePix PRO7 software.

Data normalization and statistical analyses were performed using R 2.13.1 software and the limma package [63-66]. Normalization was done by the quantile method considering the whole array data set (55 arrays). The normalized LOG_2_ of the spot-median intensity was used as the quantitative evaluation of gene expression (5.3 corresponding to background signal, 16 to spot saturation). Biological and technical repeatability were estimated higher than 87% and 96% respectively using parental strain replicate by the intra-class coefficient of correlation [67].

Comparative transcriptomic between parental strain and between disomic/normal segregants were performed with a modified t-test using the Benjamini and Hochberg false discovery rate as multiple testing correction of the t-test p-values (adjPv) [68]. The threshold used was the adjPv lower than 0.01, and a filter with a log_2_ of fold-change (logFC) greater than 0.7 or lower than −0.7 was applied for parental comparison.

Hierarchical clustering was performed using cluster v3.0 (Centered correlation and complete linkage [69]) and displayed with JavaTreeView v1.1.5r2 [70]. As previously described in Yvert *et. al.*[34], we defined a statistically significant degree of correlation between genes by permutation testing (n=10) and focused further analyses to clusters in which all pairwise correlations are greater than 0.84. At this threshold, fewer than three clusters of two genes and no cluster of more than two genes are expected by chance. Functional analysis of transcriptomic output was realized using Funspec with the Bonferroni correction at p-value cutoff of 0.05 [33]. Complete array data set is available on Gene Expression Omnibus database (global analysis: GSE41025, *PDR8* allelic switch analysis: GSE41738).

### Genotyping

The genotyping of the 14 new strains was performed with the same method than the first analysis [4]: Genomic DNA was isolated, fragmented, labeled, and hybridized on Affymetrix YGS98 microarrays, one time for each strain. A new marker map was designed doing the same selection than the precedent analysis described in Ambroset *et al.* 2011 [4] and inspired from Brem *et al.* 2002 [24]. This resulted in a map of 2140 markers.

To study genome duplication, comparative genomic hybridization (CGH-like) analysis was performed using Affymetrix normalized logarithm of PM signal of all the probes. A smoothing taking the average over 200 probes was done.

We completed the 2140 markers map with 46 markers localized in area of low markers density. These markers were based on single nucleotide polymorphism detection using Illumina veracode technology. This resulted in a good genotyping cover with an average density of 1.81 markers each 10 kbp.

### Linkage analysis

The linkage analysis was performed using R/qtl package [71] on the three datasets corresponding to the three analyses (Analysis1, Analysis2 and Analysis3). The normal model with Haley-Knott regression method was used resulting in logarithm of odds (LOD) score for each marker and pseudo-marker every 2.5 cM (7.5 kbp) (interval mapping method). An interval estimate of the location of each QTL or eQTL was obtained as the 1-LOD support interval: the region in which the LOD score is within 1 unit of the peak LOD score. For phenotypic QTLs, individual LOD score threshold for a false discovery rate of 0.05 was determined doing 1000 permutations. For eQTLs, the permutation was performed 20 times, and the average number of transcripts showing linkage at a specific LOD score threshold was used to calculate FDR.

The eQTLs closer than 40 kbp to the localization of the gene were considerate as “local” eQTLs or cis-eQTLs. This could be due to a mutation in the gene promoter (real cis-eQTL), a mutation in the protein that control its own expression as a feedback, a mutation in an other gene close that control its expression (trans-eQTL co-localized). They also can be artifacts due to mutations in sequence that reduce the mRNA affinity with the micro-array probe.

### Phylogeny and neutrality tests

To infer the evolutionary history of *WAR1* and *YRR1*, we collected their sequences from genomes available at SGD (http://www.yeastgenome.org/). All uncompleted or frameshift-containing sequences where discarded from this set. The phylogenies were inferred with MEGA [47] by the Maximum Likelihood method based on the Kimura 2-parameter model [72]. The trees with the highest log likelihood are shown. The trees are drawn to scale, with branch lengths proportional to the number of substitutions per site.

The Neutrality Index correspond to the comparison of the ratio of non synonymous to synonymous polymorphism (intra species) to the ratio of non synonymous to synonymous divergence with the nearest species. An NI lower than one reflects a paucity of non synonymous polymorphism relative to non synonymous divergence, and is indicative of positive selection; an NI greater than one indicates negative selection of deleterious alleles driving divergence between species or balancing selection. The significance of the NI test [48] was calculated using the http://bioinf3.uab.cat/mkt/MKT.asp website.

### Availability of supporting data

Sequence of 59A strain is available in http://genome.jouy.inra.fr/genyeastrait/. Transcriptomic row data are available in Gene Expression Omnibus database (global analysis: GSE41025, *PDR8* allelic switch analysis: GSE41738). Other supporting data are included in the additional files.

## Abbreviations

QTL: Quantitative trait locus; X-QTL: Extreme QTL; eQTL: Expression QTL; cis-eQTL: eQTL closer than 40 kbp to the localization of the gene (“local” eQTL); trans-eQTL: eQTL farer than 40 kbp to the localization of the gene (“distant” eQTL); aCGH: Comparative genomic hybridization in array; logFC: Log_2_ of Fold-Change; FDR: False discovery rate; LOD: Logarithm of odds; SAdM: S-adenosyl methionine; PABA: Para-amino benzoate; Rmax: Maximal fermentation rate; R70: Fermentation rate at 70% of fermentation progress; OD600: Optic density at 600 nm; Pv: P-value; adjPv: Adjusted P-value; kbp: Kilo base-pair.

## Competing interests

The authors declare that they have no competing interests.

## Author's contributions

CB participated in the design of the study, performed the data acquisition and analysis, and drafted the manuscript. CA participated in the design of the study, and realized the 59AxS288c lineage. IS performed the statistical analyses, the transcriptomic data processing and the QTL and eQTL linkages. JLL performed the phylogenic analysis and provided guidance over the data analysis and the manuscript preparation. BB conceived and coordinated the study, and provided guidance over the manuscript preparation. All authors read and approved the final manuscript.

## Supplementary Material

Additional file 1 **Example of phenotypic value distribution among the population.** Parental values are indicated in the top with open circle and black circle for 59A and S288c respectively. Heritablity (H^2^) is indicated. R70 exhibits a bimodal distribution while other phenotypes have a continuous distribution. Click here for file

Additional file 2 Clustering analysis. Click here for file

Additional file 3 **Markers map.** 2140 markers are from Affymetrix genotyping, 46 markers were based on single nucleotide polymorphism detection using Illumina veracode technology to cover area of low Affymetrix markers density. The global density is 1.81 markers each 10 kbp. Click here for file

Additional file 4 Comparison of partial disomy correction analyses. Click here for file

Additional file 5 **Hotspot 8 genes triggered by *HAP1 *** **or/and *PDR8.***
Click here for file

Additional file 6 **Flocculation phenotype triggered by *FLO1 *** **expression.** Click here for file

Additional file 7 **Phylogenic trees of the protein sequence of the two transcription factor involved in drug detoxification network variation.** Trees were drawn by MEGA5 software with the maximum likelihood method from the genome sequences available [SGD]. Parental strains are indicated by blue and yellow spot for 59A and S288c respectively. Click here for file

Additional file 8 Primer used. Click here for file
