# Supplementary figures and images for: Differential adaptation to multi-stressed conditions of wine fermentation revealed by variations in yeast regulatory networks
Source: BMC Genomics. 2013 Oct 4;14:681. doi: 10.1186/1471-2164-14-681 (PMC3870980; doi:10.1186/1471-2164-14-681)

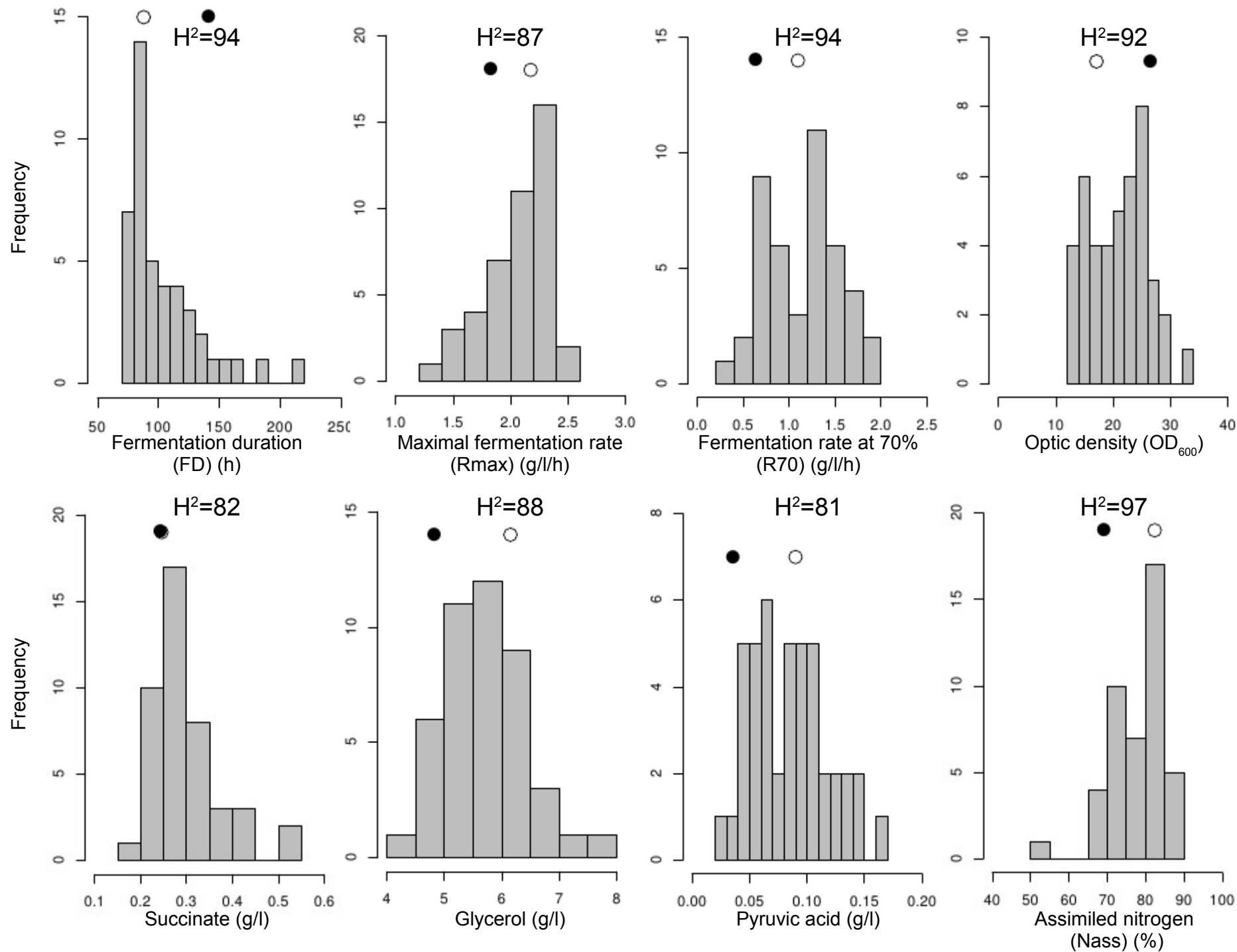

Supplement: Additional file 1 — Example of phenotypic value distribution among the population. Parental values are indicated in the top with open circle and black circle for 59A and S288c respectively. Heritablity (H2) is indicated. R70 exhibits a bimodal distribution while other phenotypes have a continuous distribution. [file 1471-2164-14-681-S1.pdf]

chromosomes

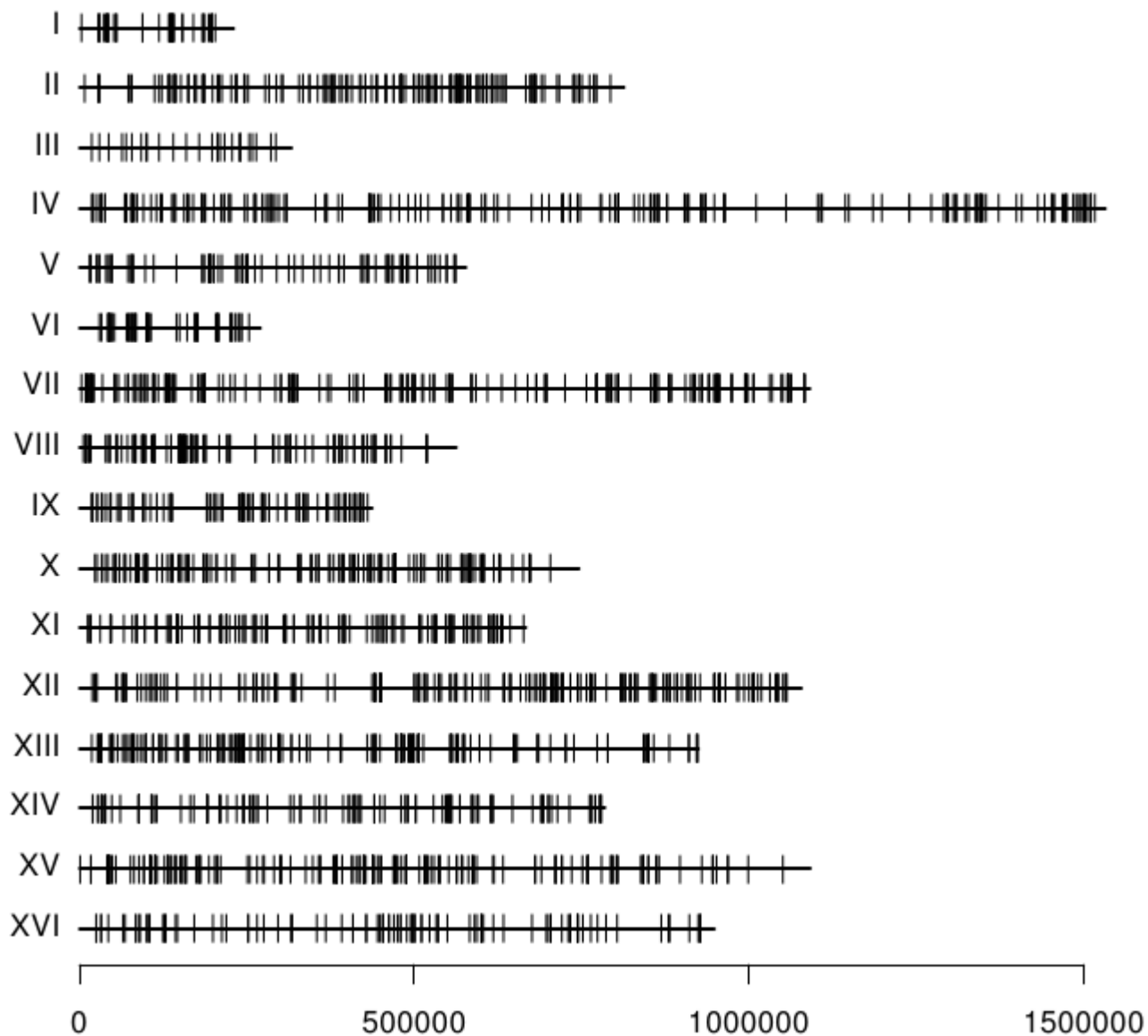

nucleotides

Supplement: Additional file 3 — Markers map. 2140 markers are from Affymetrix genotyping, 46 markers were based on single nucleotide polymorphism detection using Illumina veracode technology to cover area of low Affymetrix markers density. The global density is 1.81 markers each 10 kbp. [file 1471-2164-14-681-S3.pdf]

A

WAR1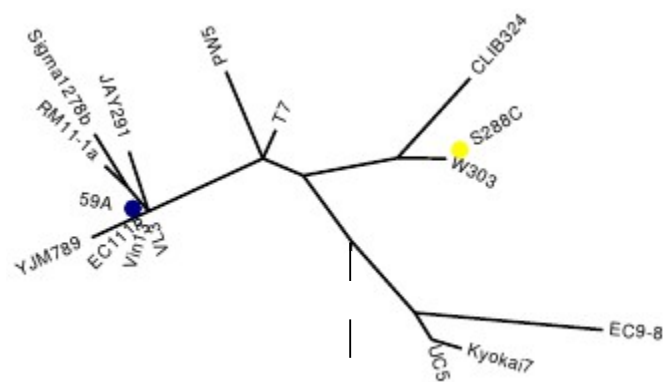

B

YRR1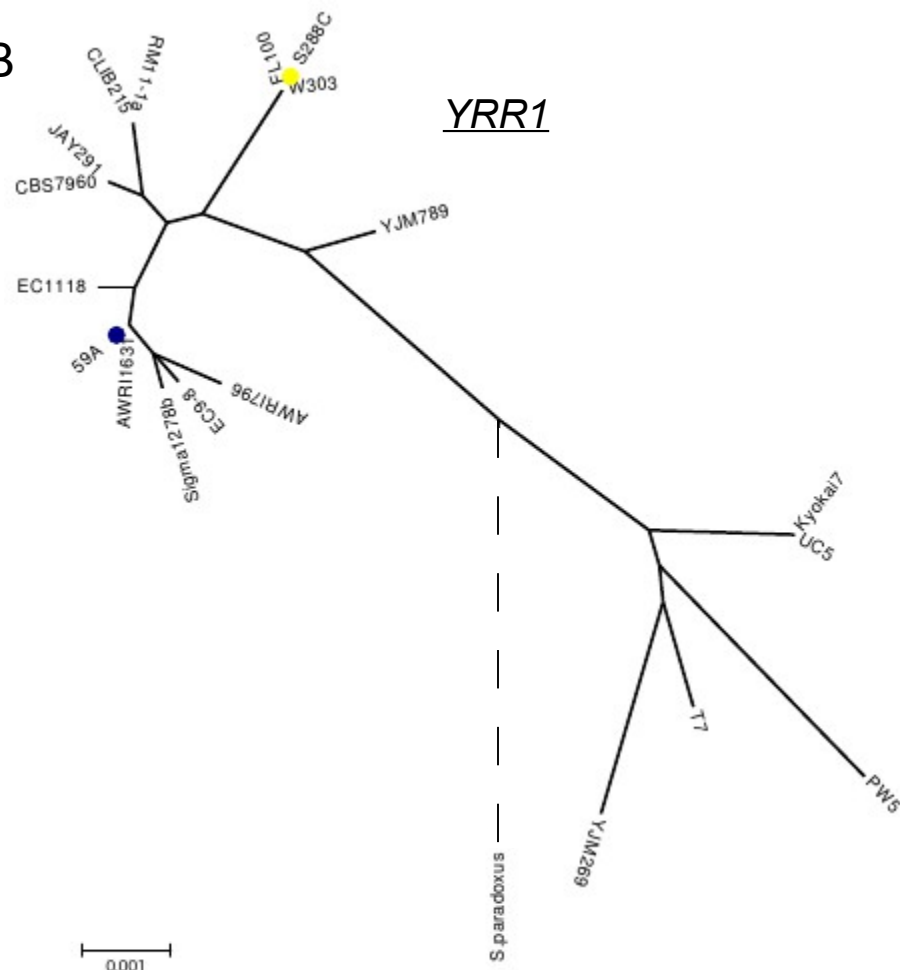

Supplement: Additional file 7 — Phylogenic trees of the protein sequence of the two transcription factor involved in drug detoxification network variation. Trees were drawn by MEGA5 software with the maximum likelihood method from the genome sequences available [SGD]. Parental strains are indicated by blue and yellow spot for 59A and S288c respectively. [file 1471-2164-14-681-S7.pdf]
