# Supplementary material for: Differential adaptation to multi-stressed conditions of wine fermentation revealed by variations in yeast regulatory networks
Source: BMC Genomics. 2013 Oct 4;14:681. doi: 10.1186/1471-2164-14-681 (PMC3870980; doi:10.1186/1471-2164-14-681)
Supplement: Additional file 2 — Clustering analysis. [file 1471-2164-14-681-S2.pdf]

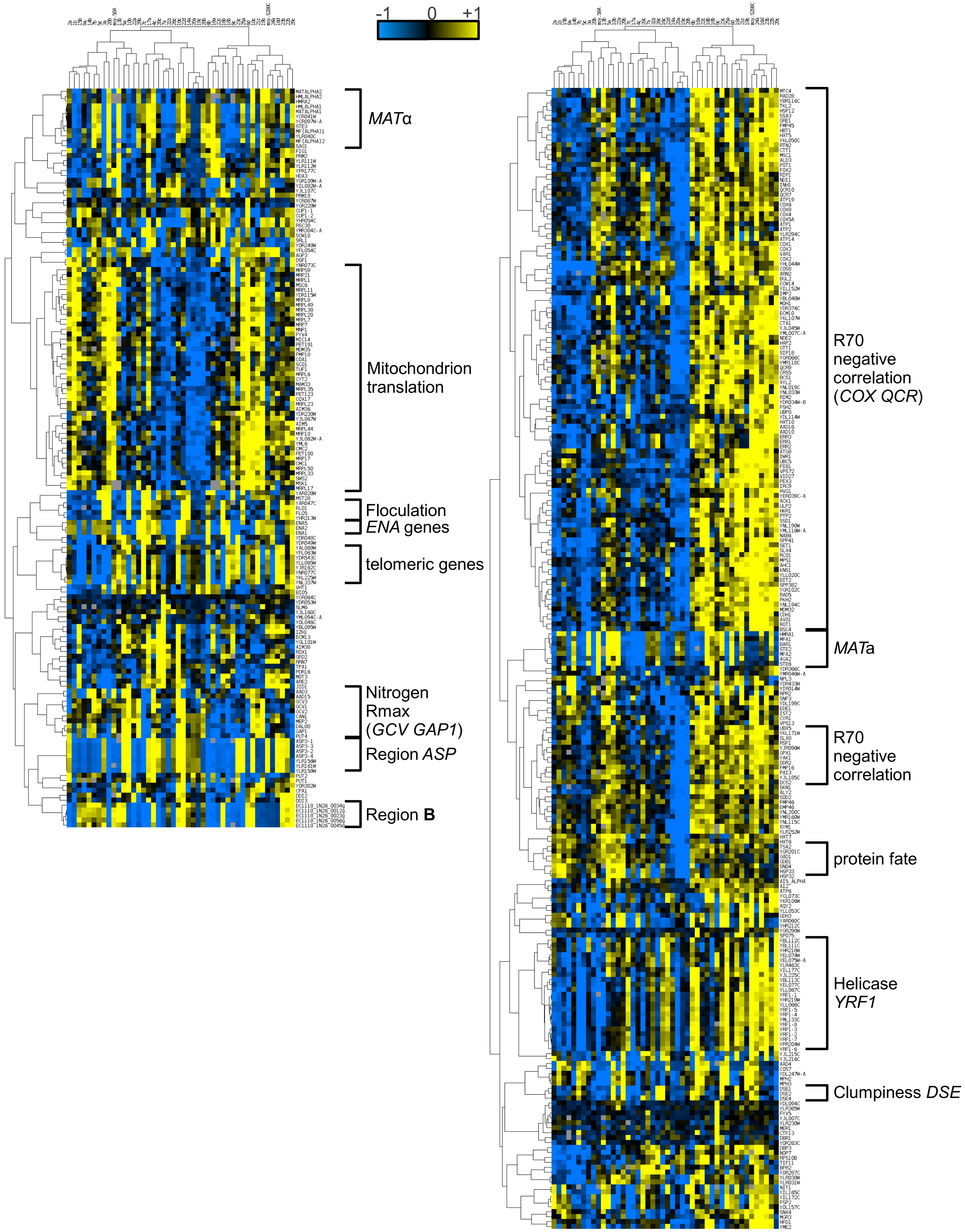
-1
0
+1
MATα
Mitochondrion translation
Floculation
ENA genes
telomeric genes
Nitrogen Rmax (GCV GAP1)
Region ASP
Region B
R70 negative correlation (COX QCR)
MATa
R70 negative correlation
protein fate
Helicase YRF1
Clumpiness DSE

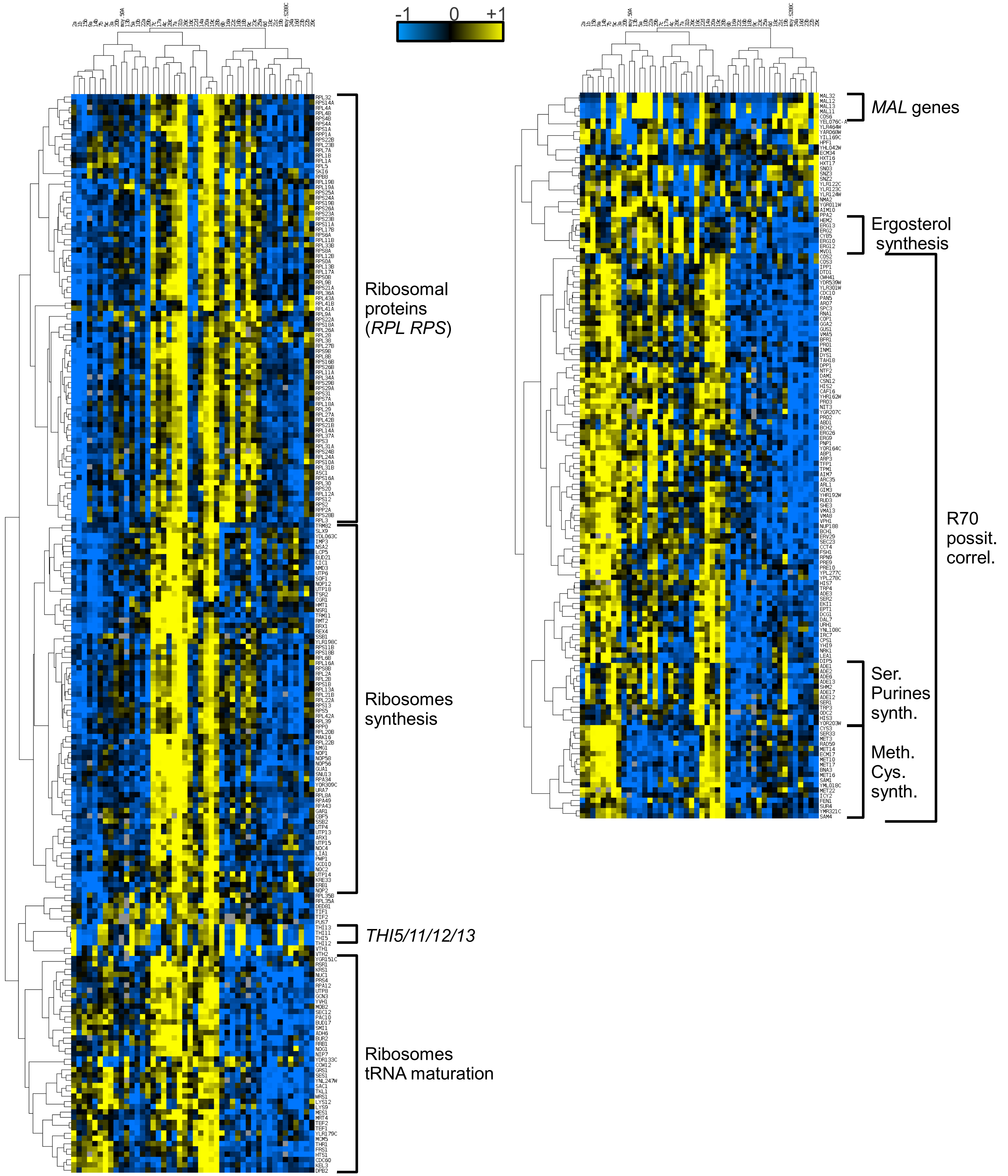

-1 0 +1
Ribosomal proteins (RPL RPS)
Ribosomes synthesis
THI5/11/12/13
Ribosomes tRNA maturation
MAL genes
Ergosterol synthesis
R70 possit. correl.
Ser. Purines synth.
Meth. Cys. synth.

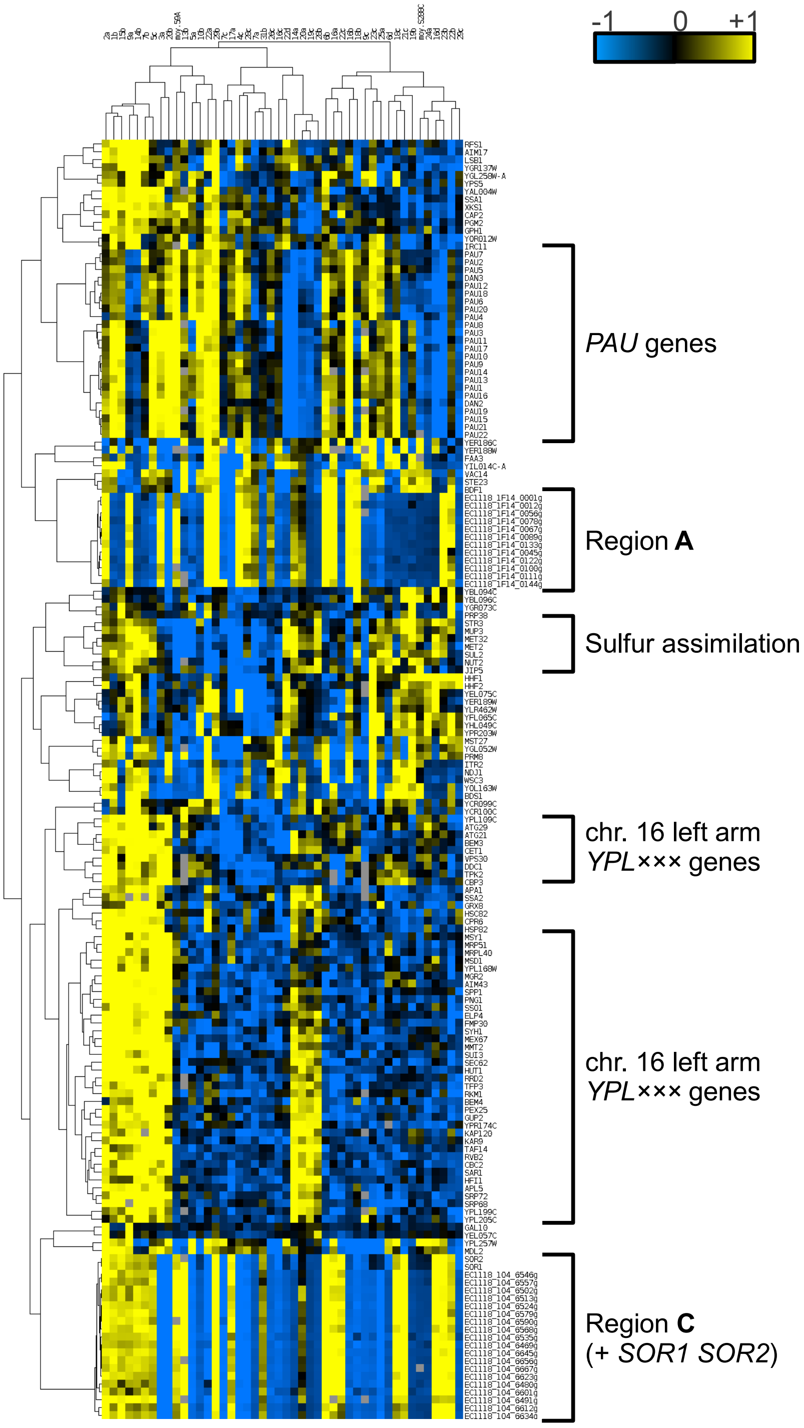
-1
0
+1
PAU genes
Region A
Sulfur assimilation
chr. 16 left arm
YPL××× genes
chr. 16 left arm
YPL××× genes
Region C
(+ SOR1 SOR2)
